# CircRNA0857 acts as an miRNA sponge to promote Newcastle disease virus replication via modulating autophagy

**DOI:** 10.1128/jvi.01121-26

**Published:** 2026-08-27

**Authors:** Siyuan Wang, Yang Qu, Weiwei Liu, Xusheng Qiu, Lei Tan, Cuiping Song, Ying Liao, Libin Chen, Chan Ding, Tao Ren

**Affiliations:** 1 College of Veterinary Medicine, South China Agricultural University 554665, Guangzhou, China; 2 Department of Avian Infectious Diseases, Shanghai Veterinary Research Institute, Chinese Academy of Agricultural Science 118161, Shanghai, China; 3 School of Agriculture and Biology, Shanghai Jiao Tong University 117750, Shanghai, China; University of Kentucky College of Medicine, Lexington, Kentucky, USA

**Keywords:** Newcastle disease virus, circular RNA, autophagy, miRNA sponge, viral replication

## Abstract

Circular RNAs (circRNAs) are a class of endogenous non-coding RNAs widely expressed across diverse organisms. These molecules are involved in various biological processes, such as transcriptional and post-transcriptional regulation, protein scaffolding, and acting as miRNA sponges. Newcastle disease virus (NDV) replicates and proliferates in a variety of cells, inducing severe organelle stress damage, autophagy, and even cell death. Previous studies have reported that NDV hijacks and uses autophagy to promote its replication and proliferation. However, the involvement and regulatory mechanisms of circRNAs in this process remain largely unexplored. The present study aimed to identify novel circRNAs that modulate NDV replication through autophagy and elucidate their underlying mechanisms. Our findings revealed that circRNA0857 was significantly upregulated in NDV-infected cells. Functional assays showed that silencing circRNA0857 markedly inhibited NDV replication, while its overexpression enhanced viral replication. Mechanistic analyses demonstrated that circRNA0857 functions as a “miRNA sponge,” specifically targeting and sequestering miR1709 and miR1746. These miRNAs were found to regulate the expression of ATG3 and ATG7, respectively, two key autophagy-related genes in the autophagy pathway. By sponging miR1709 and miR1746, circRNA0857 upregulates the expression of ATG3 and ATG7, thereby enhancing autophagy and promoting NDV infection. This study is the first to identify circRNA0857 as a critical regulator in NDV infection, elucidating its role in enhancing NDV-induced autophagy via the miR1709/ATG3 and miR1746/ATG7 signaling axes. These findings significantly advance our understanding of how circRNAs regulate viral infections and provide novel insights into the molecular mechanisms underlying NDV replication.

**IMPORTANCE:**

Circular RNAs (circRNAs), as endogenous non-coding RNAs, are widely involved in various biological processes, particularly in the regulatory modulation of miRNA functions. Newcastle disease virus (NDV) can replicate in a variety of cells and promote its proliferation by hijacking and utilizing autophagy. In this study, we screened for novel circular RNAs that regulate NDV replication through modulating host autophagy and identified circRNA0857 as a key regulator of NDV infection. It enhances autophagy and promotes viral replication by acting as a “miRNA sponge,” simultaneously targeting the two signaling axes miR1709/ATG3 and miR1746/ATG7. This research fills a gap in understanding how circRNAs regulate NDV-induced autophagy and provides a new perspective for understanding the interactions between host non-coding RNAs and viruses. This finding deepens our understanding of the pathogenic mechanisms of NDV and offers potential molecular targets for the development of antiviral strategies based on circular RNAs or miRNAs.

## INTRODUCTION

Circular RNAs (circRNAs) represent a unique class of non-coding RNAs (ncRNAs) distinguished by their covalently closed circular structure, in which the 3′ and 5′ ends are joined to form a stable loop (1–4). Unlike linear RNAs, circRNAs exhibit a longer half-life and strong resistance to ribonucleases, granting them remarkable stability and regulatory versatility within cells (5–7). The functions of circRNAs are primarily manifested through the following mechanisms. First, circRNAs have multiple binding sites for miRNAs, functioning as molecular sponges. By competitively binding to miRNAs, circRNAs modulate the expression of downstream target genes regulated by miRNAs, indirectly influencing gene expression and downstream cellular processes. In addition to their interactions with miRNAs, circRNAs possess binding sites for numerous RNA-binding proteins. This enables them to act as sponges for these proteins, further expanding their regulatory capacity (8–11). Moreover, under specific conditions, circRNAs can directly participate in protein translation. For instance, circRNAs containing internal ribosome entry sites (IRES) or modified by N6-methyladenosine (m6A) have been shown to support protein synthesis (12).

In the context of viral infections, circRNAs play critical and diverse roles in regulating infection processes. For example, during Kaposi’s sarcoma-associated herpesvirus infection, hsa_circ_0001400 exerts antiviral effects by binding to viral miR-K12-10b and inhibiting viral gene expression (13). In human immunodeficiency virus (HIV) infection, circRNAs regulate viral replication through three competitive endogenous RNA networks by modulating host immune responses, inflammatory responses, and viral infection defense mechanisms (14). Moreover, circRNAs can modulate host immune responses and cellular metabolism during viral infections. During SARS-CoV-2 infection, circRNAs regulate the expression of various factors including interleukins, epidermal growth factors, hypoxia-inducible factors, mammalian target of rapamycin (mTOR), and tumor necrosis factors. This regulation may disrupt the balance between pro-inflammatory and anti-inflammatory mechanisms, potentially leading to cytokine storms and secondary complications in COVID-19 patients (15–17). In SARS-CoV-1 infection, some circRNAs act as molecular sponges to reduce antiviral effects mediated by interferon-stimulated genes (ISGs), while others facilitate viral immune evasion and replication (18). During influenza A virus infection, circVAMP3 inhibits interactions between viral NP and RNA components (PB1, PB2, and vRNA), thereby counteracting NS1-mediated suppression of RIG-I activation and restoring host innate antiviral immunity (19). Furthermore, viruses themselves can produce circRNAs that significantly impact host cells. Epstein-Barr virus circRNAs, for instance, can bind to host miRNAs through sponging mechanisms, regulating viral replication, immune evasion, and host cell proliferation and survival (20). These findings collectively highlight the regulatory roles of circRNAs in viral pathogenesis, functioning through mechanisms spanning gene expression modulation, immune response regulation, and host-virus interaction, which provide new targets for antiviral therapy.

Autophagy, an evolutionarily conserved lysosomal degradation pathway, plays a pivotal role in maintaining cellular homeostasis through its capacity to eliminate misfolded proteins, protein aggregates, aged/damaged organelles, and intracellular pathogens (21, 22). Autophagy plays a complex dual role in the process of viral replication. On one hand, it serves as a cellular defense mechanism by degrading viral components to restrict viral replication (23). On the other hand, it can be hijacked by viruses to facilitate their own replication. Many viruses encode specific proteins to interfere with the autophagy process, such as inhibiting autophagy maturation or exploiting autophagic vesicles to provide favorable conditions for viral replication, assembly, and escape. For example, during HSV-1 infection, heterophagy facilitates the transport of viral particles to lysosomes for degradation. However, the viral protein ICP34.5 can bind to Beclin-1, thereby inhibiting autophagy and enabling the virus to evade autophagy-mediated degradation (24). Similarly, the HIV Nef protein interferes with autophagy maturation by interacting with Beclin-1, thus protecting the virus from degradation and promoting the release of mature viral particles (25). In the case of HCV infection, endoplasmic reticulum stress induces the formation of autophagosomes, but the fusion between autophagosomes and lysosomes is inhibited. This incomplete autophagy process allows HCV to utilize the autophagosome membranes for viral RNA replication, ultimately facilitating viral replication (26). The interaction between viruses and autophagy not only impacts viral pathogenicity but also offers potential targets for the development of antiviral therapeutic strategies.

Studies have shown that the interaction between autophagy and circRNA is particularly prominent in cancer and neurodegenerative diseases. CircRNA can indirectly regulate the expression of autophagy-related genes through interactions with miRNA or proteins. Furthermore, the autophagic state of the cell can also affect the production and degradation of circRNA. Under cellular stress conditions, such as oxidative stress or nutrient deprivation, enhanced autophagic activity may alter the stability and function of circRNA, thereby influencing the cellular stress response. For instance, during the development of hepatocellular carcinoma (HCC), circMDK upregulates ATG16L1 through sponge miR-346 and miR-874-3p, activates the PI3K/AKT/mTOR signaling pathway, and promotes the proliferation, migration, and invasion of HCC cells (27). In epithelial ovarian cancer, circMUC16 promotes ATG13 expression and promotes autophagy by directly binding to ATG13 (28). These studies suggest that circRNA may serve as a critical target for cancer therapy by regulating the autophagy process in tumor cells. Similarly, in viral infectious diseases, circRNAs may also influence viral replication and proliferation by modulating autophagy. Investigating this relationship could provide valuable insights and open new avenues for the discovery of innovative antiviral targets.

Newcastle disease virus (NDV) is a paramyxovirus of significant economic and medical importance. It poses a serious threat to the poultry industry and is also being explored for cancer therapy due to its oncolytic properties (29–31). Studies have shown that NDV infection is closely associated with the autophagy process in host cells, as it can hijack autophagy to create an environment favorable for viral replication. However, the specific regulatory mechanisms of circRNA in NDV-induced autophagy remain unclear. Therefore, this study aims to identify and characterize a novel circRNA involved in NDV infection and elucidate how it enhances NDV replication through autophagy. This research will highlight the regulatory role of circRNA in viral infectious diseases, providing new potential targets for antiviral therapy.

## RESULTS

### NDV infection significantly upregulated circRNA0857

**Fig 1:**
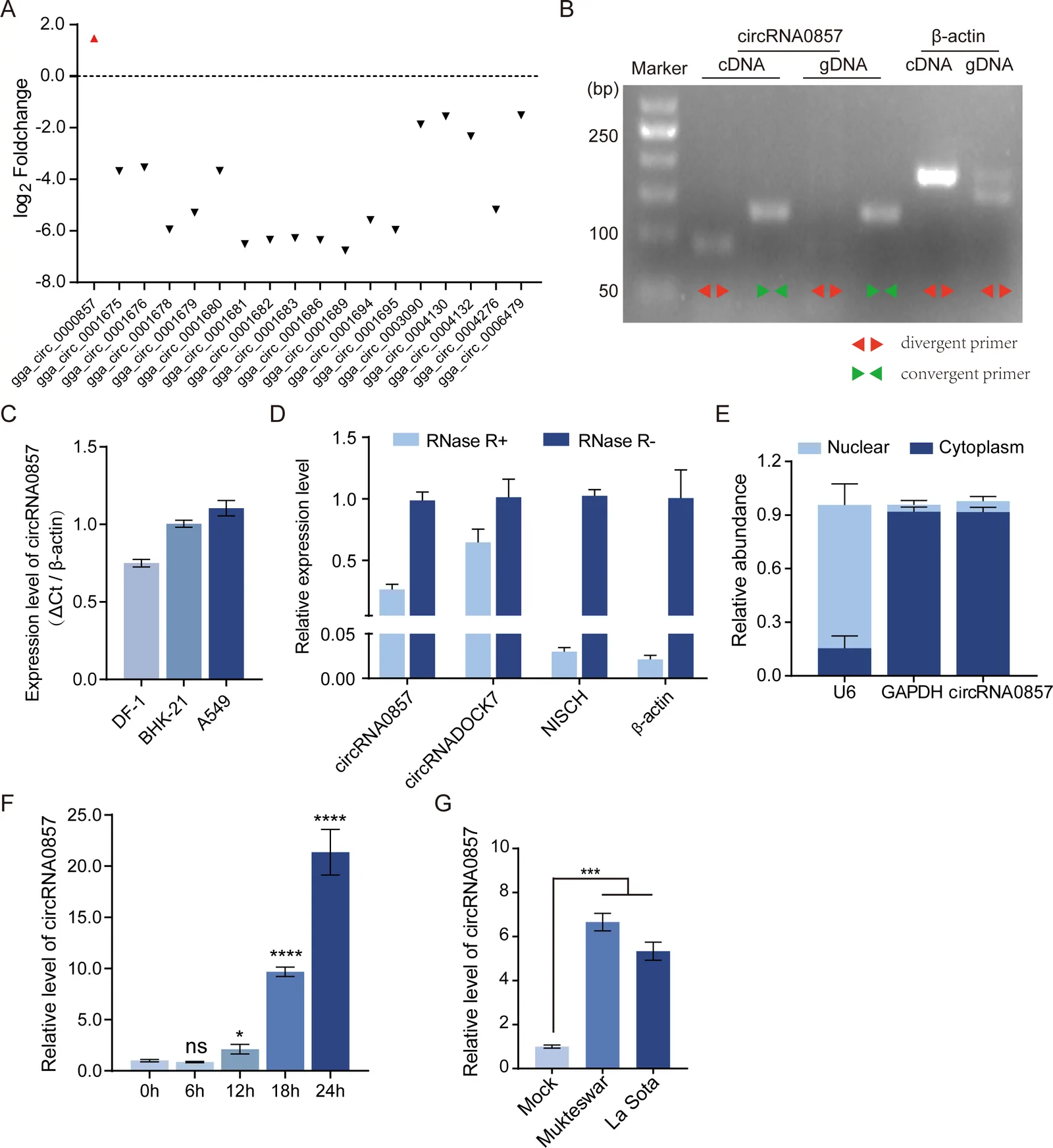
NDV infection significantly upregulated circRNA0857. (**A**) Differential expression of circRNAs in CEF cells infected by NDV. (**B**) Using divergent primers and convergent primers, PCR amplification was performed on the cDNA and gDNA of DF-1 cells, with β-actin serving as a blank control. (**C**) The relative expression abundance of circRNA0857 in DF-1 cells, BHK-21 cells, and A549 cells is represented by the ratio of △Ct values to the Ct value of β-actin. A smaller ratio indicates a higher relative expression abundance. (**D**) RNase R tolerance experiment in circRNA0857. β-actin and NISCH were used as negative controls, and known circRNADOCK7 was used as a positive control. (**E**) The nucleoplasmic separation assay was used to detect the relative levels of circRNA0857 in the nucleus and cytoplasm of DF-1 cells. (**F**) The expression levels of circRNA0857 were detected at 6, 12, 18, and 24 h after NDV infection of DF-1 cells. (**G**) The expression level of circRNA0857 was detected 24 h after DF-1 cells were infected with two different NDV strains, Mukteswar and La Sota. The multiplicity of infection (MOI) of NDV in the above experiments was set to 1. Each represents the mean ± standard deviation; *P < 0.05, ***P < 0.001 and ****P < 0.0001.

To investigate the expression patterns of circRNAs induced by NDV infection, high-throughput circRNA sequencing analysis was conducted on chicken embryo fibroblasts (CEFs) infected with NDV for 12 h and uninfected controls, with three independent biological replicates for each condition. These sequencing results were based on prior transcriptomic information obtained from CEFs infected with the Herts/33 strain using RNA-sequencing technology (32, 33). Bioinformatics analysis of the sequencing data indicated that circRNAs in CEF cells, regardless of NDV infection, were primarily composed of exonic circRNAs, intronic circRNAs, and intergenic circRNAs, with intronic circRNAs showing a significantly higher abundance (Fig. S1A). Quantitative normalization and differential expression analysis of known and newly discovered circRNA expression levels were conducted. After NDV infection, the expression of 18 circRNAs changed, with 17 showing downregulation and 1 showing upregulation (Fig. 1A; Table 1). We further analyzed and identified the only upregulated circRNA, gga_circ_0000857 (circRNA0857). Further investigation of the relative abundance of circRNA0857 revealed that, compared to its expression in human-derived A549 cells and hamster-derived BHK-21 cells, circRNA0857 exhibits a higher relative abundance in avian-derived DF-1 cells (Fig. 1C). Due to the closed circular structure characteristic of circRNA, only the back splice junction-sequence (BSJ-S) of circRNA is amplified in cDNA, and it is not amplified in gDNA. Consistent with our expectations, we successfully amplified bands in the cDNA of DF-1 cells using divergent primers and convergent primers, whereas bands were only amplified in gDNA using convergent primers (Fig. 1B). Further Sanger sequencing of the amplified bands and comparison with the UCSC Genome Browser indicated that circRNA0857 is a circular structure composed of 341 nucleotides, derived from the 14th, 15th, and 17th exons of the NISCH gene (Fig.S1B and C). NISCH encodes nischarin, a multifunctional scaffold protein involved in cell migration regulation and signaling pathways including Rac and AMPK. The circular structure confers high stability to circRNA0857, enabling it to resist digestion by the RNase R exonuclease. As expected, with the known stable circular RNA circDOCK7 as a positive control (34), circRNA0857 demonstrated significantly greater resistance to digestion compared to the mRNA of NISCH and β-actin (Fig. 1D). These results confirm that circRNA0857 is a correctly circularized circRNA. By isolating the nuclear and cytoplasmic components of DF-1 cells, we further elucidated the subcellular localization of circRNA0857. The results indicate that circRNA0857 is predominantly located in the cytoplasm (Fig. 1E). Subsequently, we further confirmed the impact of NDV infection on the expression of circRNA0857. The results showed that with the extension of the virus infection time, the expression level of circRNA0857 increased (Fig. 1F). The mesogenic (Mukteswar) and avirulent (LaSota) strains also induced similar upregulation of circRNA0857 (Fig. 1G), indicating a general host response rather than a virulence-specific event. These results indicate that circRNA0857 is expressed in avian DF-1 cells and is able to correctly form a circular structure. NDV infection significantly promotes the upregulation of circRNA0857 expression in DF-1 cells.

**TABLE 1:** BSJ sequence of circRNA

	circRNA	Log_2_ fold change	BSJ sequence
1	gga_circ_0000857	1.5679	TGAATTCTTCCATGGCAACATTGCAGAGAAATAGTTTGTGAAGAAACAGTCCTAGC
2	gga_circ_0001675	−3.6789	TGAACGTGTGCAGCTCCTCCATACCCAGAACACCAGCTTGATCAACACCAAGAAGA
3	gga_circ_0001676	−3.5365	CCTGAGAAACACACAAGGAGTGCTCAAGGATACCCAGATACACTTGGACGATGCTC
4	gga_circ_0001678	−5.9478	GGACAAGAAGCAGAAGAACTTTGACAAGATCCTGTCGGAGTGGAAGCAGAAGTACG
5	gga_circ_0001679	−5.2946	AGCGCACAGAGGAGCTGGAGGAGGCCAAGAAGAAGCTGGCACAGCGCCTGCAAGAT
6	gga_circ_0001680	−3.667	CAGAAAGCTCGTCTACAAACAGAGTCAGGTGAATATTCACGCCAGGTGGAGGAGAA
7	gga_circ_0001681	−6.5149	AACAGCAGCTGGATGAGAAACTGAAGAAGAAAGACTTTGAAATCAGCCAGATCCAG
8	gga_circ_0001682	−6.3489	AACAGCAGCTGGATGAGAAACTGAAGAAGAAAGACTTTGAAATCAGCCAGATCCAG
9	gga_circ_0001683	−6.2771	CACCCAGTACAAATTTGGTCACACCAAGGTGTTCTTCAAAGCTGGGCTGCTGGGAC
10	gga_circ_0001686	6.3509	GGCTGCCTGCATTGAGCTCATTGAAAAGCCCATGGGCATCTTCTCCATCCTGGAAG
11	gga_circ_0001689	−6.7634	GGACATCGCTGGCTTTGAGATCTTTGATTTCAACAGCCTGGAGCAGCTGTGCATCA
12	gga_circ_0001694	−5.582	GGAGAAGGCCAAGAAGGCCATCACCGATGCAGCCATGATGGCAGAAGAGCTGAAGA
13	gga_circ_0001695	−5.9639	GGAGAAGGCCAAGAAGGCCATCACCGATGCAGCCATGATGGCAGAAGAGCTGAAGA
14	gga_circ_0003090	−1.8721	ACCGGACTGGCTTCACCTTCAAATACAGGGATGAGCTCAAGCGGCACTACAACCTG
15	gga_circ_0004130	−1.5517	GATCGACCTGCAGTCCCAGCTCAGCACGAAAGCGGCGCTGGAGGGCACCCTGGCCG
16	gga_circ_0004132	−2.3287	GGCTGGCAGCTGATGACTTCAGAACCAAGTTTGAGACGGAGCAGGCCCTGCGTCTG
17	gga_circ_0004276	−5.1791	CACAAGATTAATCAATGTCAACTGACAGAAACCCAGATTCTAGAAACAACCAATTG
18	gga_circ_0006479	−1.512	ACGGGAAGGATCTGAAGGAGAGAGCCCTAATTGACATGTATGTGGAAGGACTGGCA

### CircRNA0857 promoted NDV proliferation

**Fig 2:**
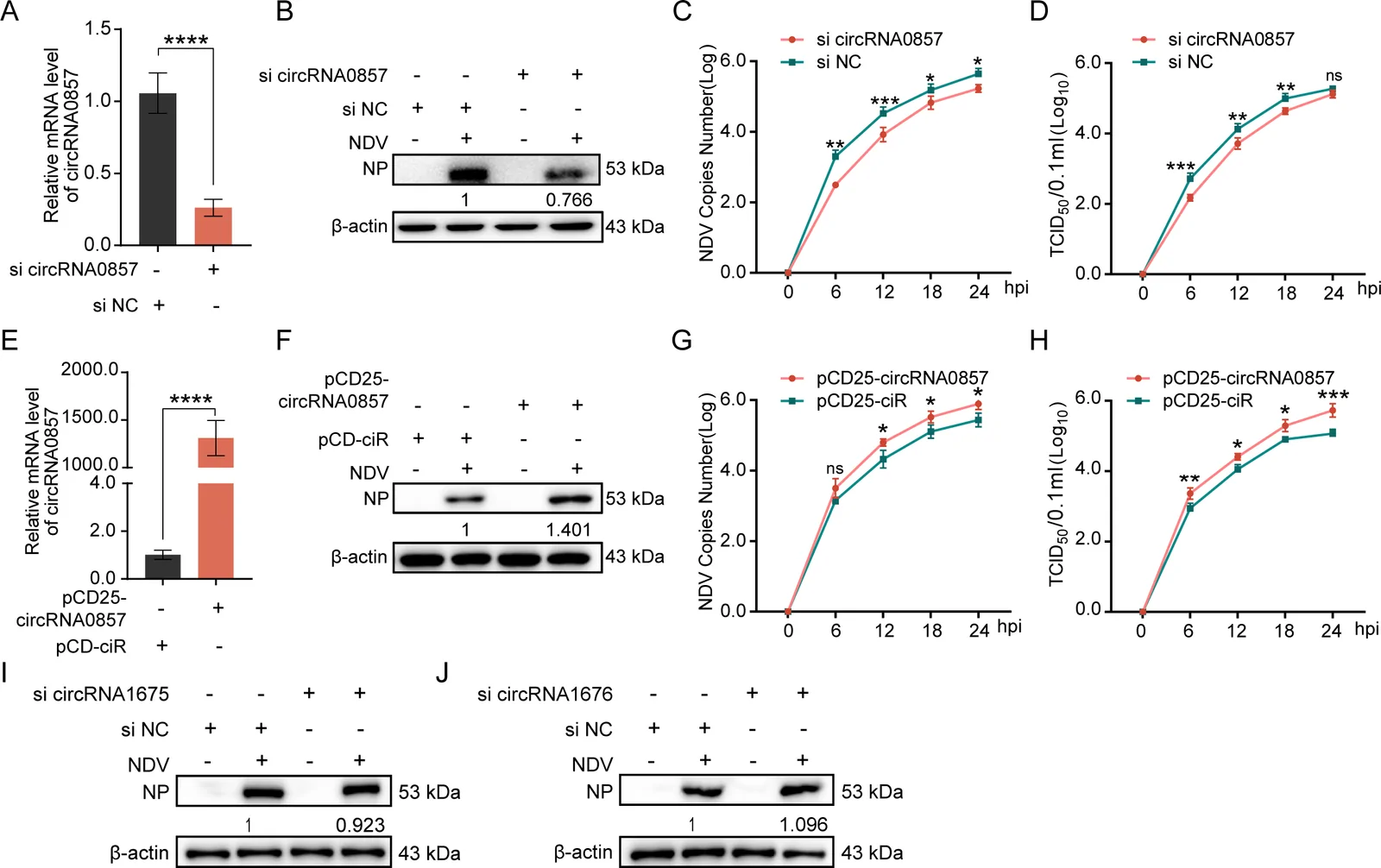
CircRNA0857 promoted NDV proliferation. (**A and E**) DF-1 cells were transfected with sicircRNA0857 or overexpressed plasmid (pCD25-circRNA0857). qRT-PCR analysis of circRNA0857 levels and then infected with NDV. (**B and F**) Cells were harvested at 12 h post-infection, western blot analyses of NDV-NP protein level. (**C and G**) Cells were harvested at 0, 6, 12, 18, and 24 h post-infection. The viral copy number was determined by qRT-PCR. (**D and H**) Cell culture supernatants were harvested at 0, 6, 12, 18, and 24 h post-infection for the determination of virus titer. The data are from three independent experiments. (**I and J**) DF-1 cells were transfected with sicircRNA1675 or sicircRNA1676 for 48 h and then infected with NDV. Cells were harvested at 12 h post-infection, western blot analyses of NDV-NP protein level, and grayscale analysis performed. The MOI of NDV in the above experiments was set to 1. Each represents the mean ± standard deviation; *P < 0.05, **P < 0.01, ***P < 0.001, and ****P < 0.0001.

NDV infection was found to significantly upregulate the expression of circRNA0857, leading us to hypothesize that this upregulation may play a role in regulating viral replication. To test this hypothesis, we modulated circRNA0857 levels in DF-1 cells through silencing and overexpression and evaluated the subsequent impact on NDV replication. First, we used small interfering RNA (siRNA) targeting circRNA0857 to effectively reduce its expression levels in cells (Fig. 2A). The effects of circRNA0857 knockdown on NDV infection were assessed by measuring the mRNA and protein levels of the NDV nucleoprotein (NP) as well as extracellular viral yields at various time points post-infection. Specifically, silencing circRNA0857 significantly reduced the protein levels of the NDV NP (Fig. 2B), viral copy number (Fig. 2C), and viral titer in the cell supernatant (Fig. 2D) at 6, 12, 18, and 24 h post-infection. To further confirm these, corroborate, and minimize potential off-target effects associated with siRNA transfection, intracellular expression levels of circRNA0857 were increased by transfecting an overexpressed plasmid (Fig. 2E). Overexpression of circRNA0857 in DF-1 cells enhanced NDV replication, as demonstrated by higher NDV NP protein levels (Fig. 2F), increased viral copy number (Fig. 2G), and elevated viral titers in the cell supernatant (Fig. 2H). To verify the specificity of this effect, we selected two downregulated circRNAs (circRNA1675 and circRNA1676) identified from our RNA-seq data and examined their effects on NDV replication. Knockdown of either circRNA did not significantly affect NP protein expression (Fig. 2I and J), confirming that the pro-viral function is specific to circRNA0857. Together, these results indicate that circRNA0857 plays a supportive role in promoting NDV replication.

### CircRNA0857 regulated autophagy via targeting miR1709 and miR1746

**Fig 3:**
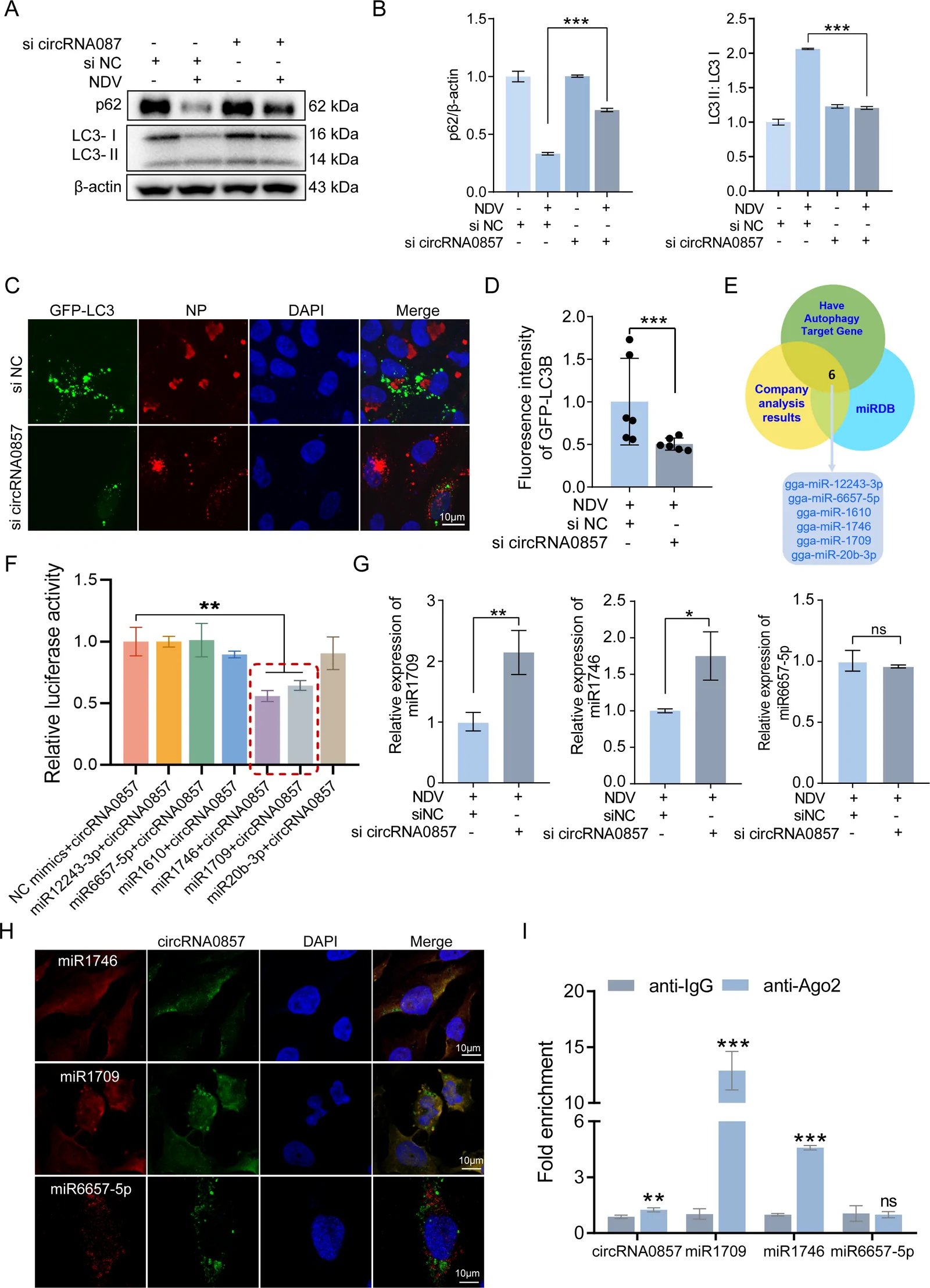
CircRNA0857-regulated NDV-induced autophagy via targeting miR1709 and miR1746. (**A and B**) DF-1 cells were transfected with sicircRNA0857 for 48 h and then infected with NDV, and cells were harvested at 24 h post-infection. Western blot analyses of the LC3 and p62 protein levels, and grayscale analysis performed. (**C and D**) DF-1 cells were co-transfected with sicircRNA0857 or siNC and GFP-LC3B for 48 h, then infected with NDV, and LC3 spots were observed and quantified by immunostaining 18 h post-infection. Scale bars: 10 μm. (**E**) miRNAs adsorbed by circRNA0857 and targeting autophagy genes were obtained by intersecting three databases. (**F**) miRNA mimics and pmirGLO-circRNA0857 were co-transfected into HEK-293T cells for 36 h and then infected with NDV. Cells were harvested to detect luciferase activity. (**G**) DF-1 cells were transfected with sicircRNA0857 or siNC for 48 h and then infected with NDV, qRT-PCR analyses of the mRNA levels of miR1709, miR1746, and miRNA6657-5p. (**H**) The co-localization of circRNA0857 with miR1709, miR1746, or miRNA6657-5p was detected using fluorescence *in situ* hybridization. Scale bars: 10 μm. (**I**) Determine the enrichment levels of circRNA0857, miR1709, and miR1746 combined with AGO2 relative to IgG through RNA immunoprecipitation assay. The MOI of NDV was set at 1 in all the experiments mentioned above. Each bar represents the mean ± standard deviation; **P < 0.01, ***P < 0.001, and ****P < 0.0001; ns, not significant.

Our previous studies have confirmed that cellular autophagy facilitates the proliferation of NDV (35). Considering the extensive involvement of circRNA in the autophagy process, we hypothesize that the enhancement of NDV replication by circRNA0857 may depend on the occurrence of autophagy. Consistent with our expectations, silencing circRNA0857 significantly inhibited NDV-induced autophagy, as evidenced by the reduction in the conversion of LC3-I to LC3-II and the accumulation of p62 (Fig. 3A and B). Conversely, overexpression of circRNA0857 promoted p62 degradation and LC3-II conversion in NDV-infected cells, and treatment with chloroquine (CQ), an autophagy inhibitor that blocks autophagosome-lysosome fusion, reversed these effects and led to a marked accumulation of LC3-II (Fig. S2A and B). Furthermore, immunofluorescence analysis confirmed that silencing circRNA0857 significantly reduced NDV-induced LC3 puncta formation (Fig. 3C and D). In summary, these results suggest that circRNA0857 regulates NDV-induced autophagy.

Given that circRNAs primarily function as molecular “sponges” that adsorb miRNAs, thereby alleviating their inhibitory effects on target genes, we conducted comprehensive screening of autophagy-related miRNAs to elucidate the molecular mechanism underlying circRNA0857-mediated autophagy regulation. Through cross-analysis of prediction results from specialized databases, including miRDB and autophagy target gene-related databases, we identified six candidate miRNAs potentially interacting with circRNA0857: miR12243-3p, miR6657-5p, miR1610, miR1709, miR1746, and miR20a-3p (Fig. 3E). To further investigate the interactions between these miRNAs and circRNA0857, we constructed a dual-luciferase reporter plasmid containing the circRNA0857 fragment (pmirGLO-circRNA0857). Dual-luciferase reporter assays revealed that luciferase activity significantly decreased in cells co-transfected with miR1709 or miR1746 and pmirGLO-circRNA0857, compared to cells transfected with negative control (NC) mimics. In contrast, the other four miRNAs did not significantly affect luciferase activity (Fig. 3F). We further examined the effect of NDV infection on the expression of miR1709 and miR1746. The results showed that miR1709 and miR1746 were mildly downregulated upon NDV infection (Fig. S2C). Additionally, silencing circRNA0857 was found to upregulate the expression levels of miR1709 and miR1746, but not the unrelated control miRNA miR-6657-5p (Fig. 3G). To further validate the interaction between miR1709, miR1746, and circRNA0857, we confirmed the co-localization of circRNA0857 with miR1709 and miR1746 through fluorescence *in situ* hybridization experiments, but miR-6657-5p did not show co-localization with circRNA0857 under the same conditions (Fig. 3H). Given that Argonaute 2 (AGO2) protein binds to miRNA to form the RNA-induced silencing complex, which mediates the interaction between circRNA and miRNA, we further conducted RNA immunoprecipitation (RIP) assays using AGO2-specific antibodies. The results demonstrated that circRNA0857, miR1709, and miR1746 were significantly enriched in the AGO2-specific antibody group compared to the IgG non-specific antibody group, whereas the unrelated control miRNA miR-6657-5p showed no significant enrichment (Fig. 3I). Collectively, these findings indicate that circRNA0857 regulates autophagy by functioning as a molecular “sponge” for miR1709 and miR1746, thereby modulating their inhibitory effects on autophagy-related genes.

### miR1709 and miR1746 targeted ATG3 and ATG7, respectively

**Fig 4:**
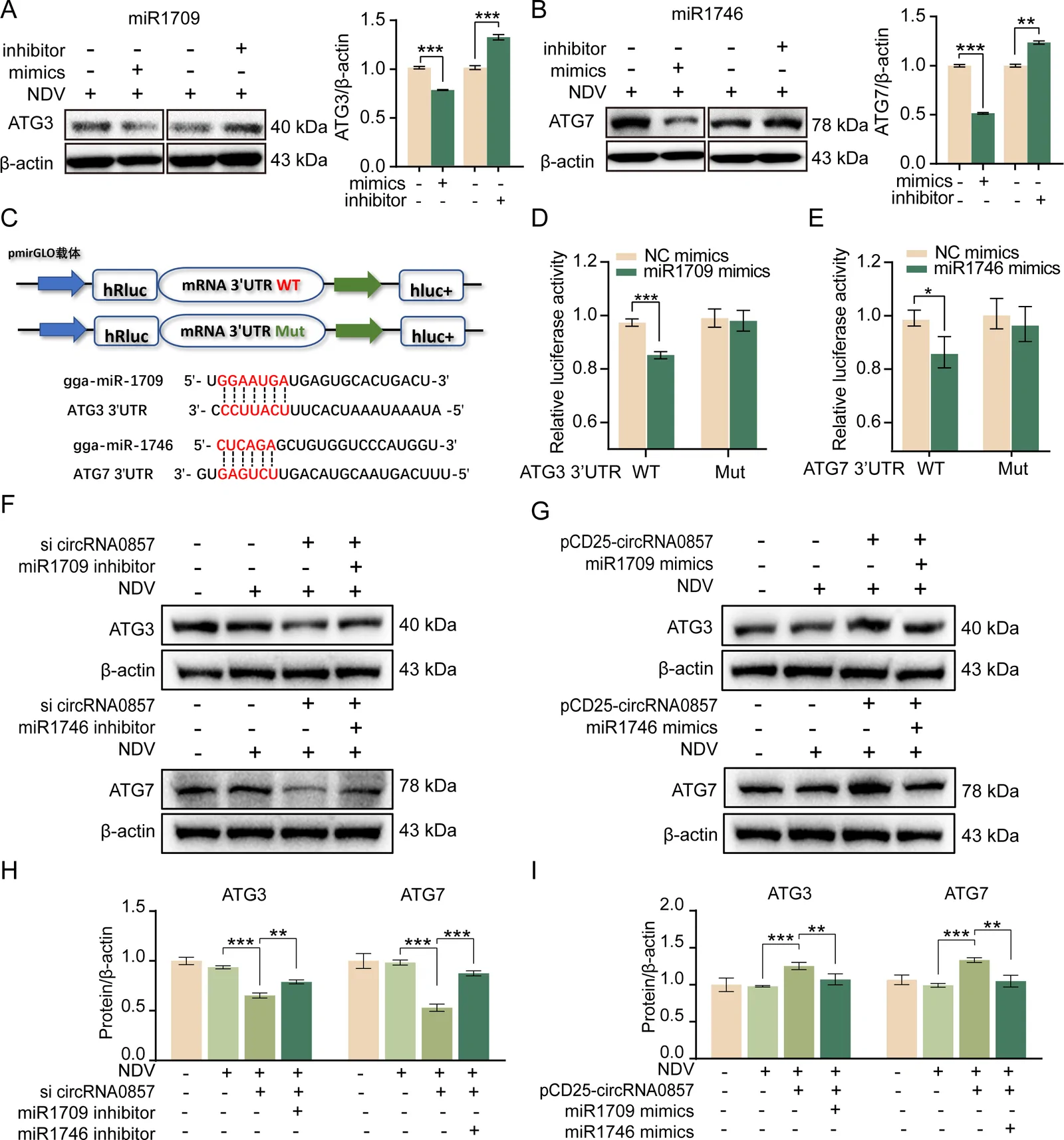
miR1709 and miR1746 targeted ATG3 and ATG7, respectively. (**A and B**) DF-1 cells were transfected with mimics or inhibitors of miR1709 or miR1746 for 48 h, then infected with NDV. Western blot analyses of ATG3 and ATG7 protein levels, and grayscale analysis performed. (**C**) The binding sites of miR1709 and ATG3 3′UTR and miR1746 and ATG7 3′UTR were predicted by RNA22 v2 prediction website. Then constructed the dual-luciferase reporter plasmids (pmirGLO-ATG3 3′UTR WT, pmirGLO-ATG3 3′UTR Mut, pmirGLO-ATG7 3′UTR WT, and pmirGLO-ATG7 3′UTR Mut). (**D**) HEK-293T cells were transfected with pmirGLO-ATG3 3′UTR WT or pmirGLO-ATG3 3′UTR Mut and miR1709 or NC mimic for 36 h, then infected with NDV. Luciferase activity was detected by DLR. (**E**) HEK-293T cells were co-transfected with pmirGLO-ATG7 3′UTR WT or pmirGLO-ATG7 3′UTR Mut and miR1746 or NC mimic for 36 h, then infected with NDV. Luciferase activity was detected by DLR. (**F and H**) DF-1 cells were transfected with sicircRNA0857 + NC inhibitor, sicircRNA0857 + miR1709, or miR1746 inhibitor for 48 h, then infected with NDV. Western blot analyses of ATG3 or ATG7 protein levels (**F**), and grayscale analysis performed (**H**). (**G and I**) DF-1 cells were transfected with pCD25-circRNA0857 + NC mimics, pCD25-circRNA0857 + miR1709, or miR1746 mimics for 48 h, then infected with NDV. Western blot analyses of ATG3 or ATG7 protein levels (**G**), and grayscale analysis performed (**I**). The MOI of NDV was set at 1 in all the experiments mentioned above. Each bar represents the mean ± standard deviation; *P < 0.05, **P < 0.01 and ***P < 0.001; ns, not significant.

The aforementioned results have confirmed that circRNA0857 enhances autophagy by adsorbing miR1709 and miR1746. It is essential to further identify the downstream autophagy-related target genes of miR1709 and miR1746. According to predictions from the miRDB database, ATG3 and ATG7 have been identified as autophagy-related target genes for miR1709 and miR1746, respectively. miRNAs typically regulate their target mRNAs by destabilizing them or inhibiting their translation. To investigate the regulatory roles of miR1709 and miR1746 in modulating ATG3 and ATG7 expression, we first assessed the impact of miR1709 and miR1746 mimics (to enhance miRNA activity) and inhibitors (to suppress endogenous miRNA function) on the mRNA levels of these target genes. The results showed that neither overexpression nor inhibition of miR1709 and miR1746 resulted in significant alterations in ATG3 or ATG7 mRNA levels (Fig. S2D), indicating that the suppression of ATG3 and ATG7 by miR1709 and miR1746 is not mediated through mRNA degradation or destabilization. Specifically, overexpression of miR1709 and miR1746 mimics led to a pronounced reduction in ATG3 and ATG7 protein abundance, respectively, whereas these miRNA inhibitors significantly rescued ATG3 and ATG7 protein expression (Fig. 4A and B). To further confirm that ATG3 and ATG7 are target genes of miR1709 and miR1746, we performed a dual-luciferase reporter assay. The RNA22 v2 prediction website predicted miR1709 and miR1746 binding sites in the 3′UTRs of ATG3 and ATG7, respectively. Subsequently, we constructed dual-luciferase reporter plasmids containing either wild-type (WT) or mutant (Mut) versions of these 3′UTRs: pmirGLO-ATG3 3′UTR WT, pmirGLO-ATG3 3′UTR Mut, pmirGLO-ATG7 3′UTR WT, and pmirGLO-ATG7 3′UTR Mut (Fig. 4C). Luciferase assays revealed that co-transfection of miR1709 with pmirGLO-ATG3 3′UTR WT significantly reduced luciferase activity compared to co-transfection with NC mimics (Fig. 4D). A parallel effect was observed for miR1746, which markedly reduced luciferase activity in cells harboring the ATG7 3′UTR WT construct (Fig. 4E). These data conclusively identify ATG3 and ATG7 as direct targets of miR1709 and miR1746, respectively. Additionally, silencing circRNA0857 significantly reduced ATG3 and ATG7 expression; this suppression was rescued by co-treatment with miR1709 and miR1746 inhibitors (Fig. 4F and H). Moreover, circRNA0857 overexpression promoted ATG3 and ATG7 expression in NDV-infected cells (Fig. 4G and I). These results indicate that circRNA0857 functions as a competing endogenous RNA to sequester miR1709 and miR1746, thereby counteracting their repression of autophagy-related targets.

### miR1709 and miR1746 inhibited NDV-induced autophagy

**Fig 5:**
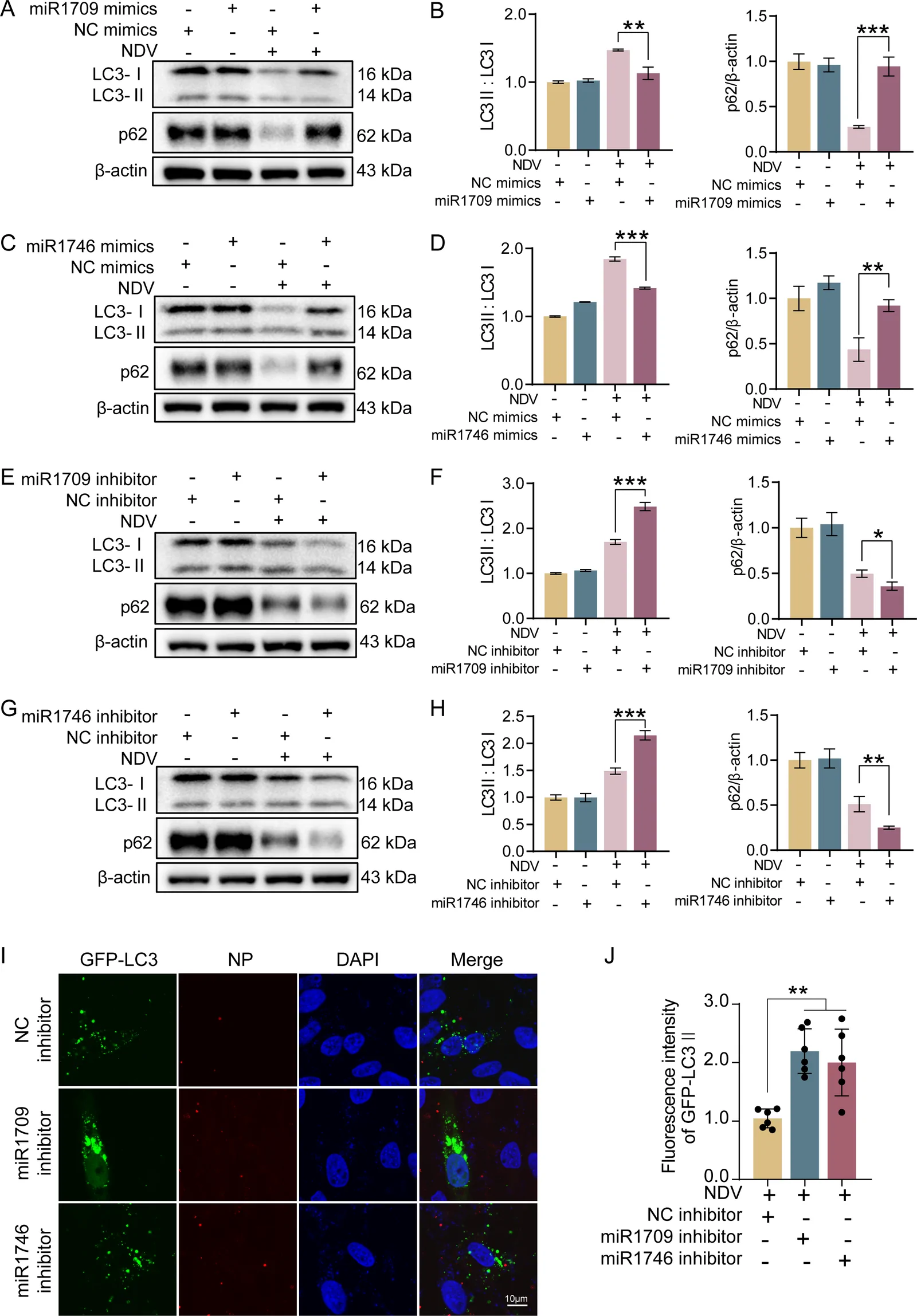
miR1709 and miR1746 inhibited NDV-induced autophagy. (**A–H**) DF-1 cells were transfected with the mimics or inhibitors of miR1709 or miR1746 for 48 h and then infected with NDV. Western blot analyses of the LC3 and p62 protein levels, and grayscale analysis performed. (**I and J**) DF-1 cells were transfected with NC, miR1709 or miR1746 inhibitors and GFP-LC3, respectively, then infected with NDV. Immunostaining was performed at 18 h post-infection, LC3 spots were observed and quantified by immunostaining. Scale bars: 10 μm. The MOI of NDV was set at 1 in all the experiments mentioned above. Each bar represents the mean ± standard deviation; *P < 0.05, **P < 0.01, and ***P < 0.001.

Since ATG3 and ATG7 are critical mediators of LC3-I lipidation into LC3-II and central regulators of the autophagy machinery, we first verified the roles of ATG3 and ATG7 in NDV-induced autophagy. The results revealed that targeted silencing of ATG3 and ATG7 substantially attenuated LC3-I to LC3-II conversion while inducing p62 accumulation (Fig. S3A through D). Consistent results were obtained in immunofluorescence experiments, where silencing ATG3 and ATG7 resulted in a reduction of LC3 puncta formation (Fig. S3E and F). The aforementioned results have confirmed that miR1709 and miR1746 inhibit NDV-induced autophagy by targeting ATG3 and ATG7, respectively. Subsequently, the effects of miR1709 and miR1746 on autophagic flux were examined. As expected, miR1709 and miR1746 mimics significantly suppressed the conversion of LC3-I to LC3-II and promoted the accumulation of p62 (Fig. 5A through D). Conversely, inhibitors of miR1709 and miR1746 enhanced the conversion of LC3-I to LC3-II and reduced p62 accumulation (Fig. 5E through H). These findings were further corroborated by immunofluorescence experiments, which demonstrated that miR1709 and miR1746 inhibitors enhanced NDV-induced LC3 puncta formation, indicating increased autophagosome formation (Fig. 5I and J). These data collectively establish a regulatory axis, where miR1709/ATG3 and miR1746/ATG7 regulate NDV-driven autophagy.

To demonstrate the broad role of miR1709 and miR1746 in regulating autophagy, we examined the effects of these miRNAs on autophagy flux under both starvation-induced and rapamycin-treated conditions. Cells were cultured in Earle’s Balanced Salt Solution (EBSS) to simulate nutrient deprivation. The results indicated that both miR1709 and miR1746 mimics inhibited autophagy flux (Fig. S4A and B), while their respective inhibitors promoted autophagy (Fig. S4C and D). Similar results were observed in rapamycin-induced autophagy (Fig. S4E and H). These findings suggest that miR1709 and miR1746 consistently modulate autophagy across different induction conditions. Notably, when all external factors inducing cellular autophagy are removed, neither the mimics nor the inhibitors of miR1709 and miR1746 exert a significant regulatory effect on autophagy flux (Fig. S5). These results imply that miR1709 and miR1746 are specifically activated in response to external factors that induce autophagy, such as starvation, rapamycin treatment, or NDV infection.

### miR1709/ATG3 and miR1746/ATG7 inhibited viral replication by suppressing autophagy

**Fig 6:**
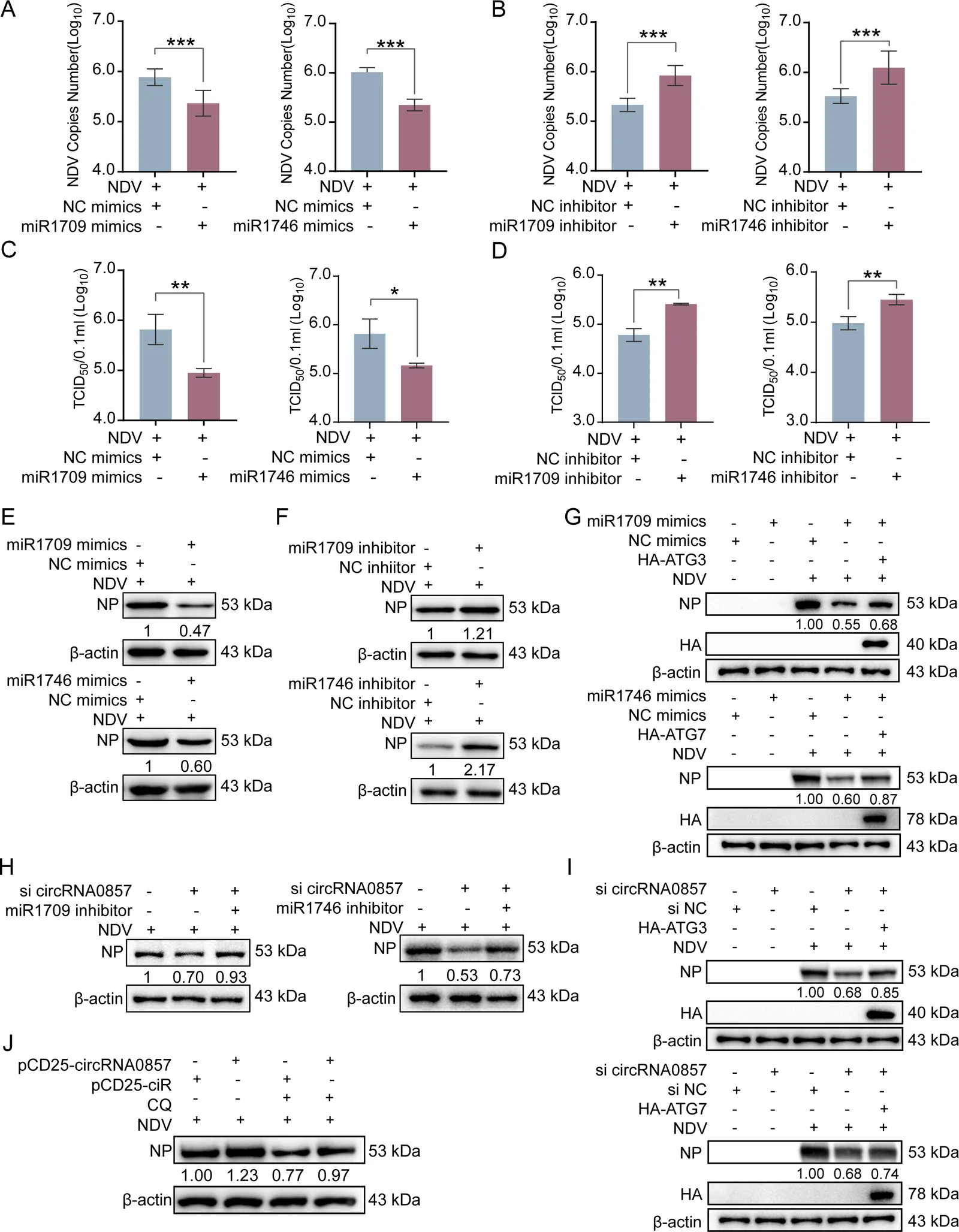
miR1709/ATG3 and miR1746/ATG7 inhibited viral replication by suppressing autophagy. (**A–F**) DF-1 cells were transfected with the mimics or inhibitors of miR1709 or miR1746 for 48 h and then infected with NDV. (**A and B**) Cells were harvested at 12 h post-infection. The NDV copy number was determined by qRT-PCR. (**C and D**) Cell culture supernatants were harvested at 12 h post-infection for the determination of virus titer. (**E and F**) Cells were harvested at 12 h post-infection, western blot analyses of NDV-NP protein level, and grayscale analysis performed. The data are from three separate experiments. (**G**) DF-1 cells were transfected with miR1709 mimics and HA-ATG3, miR1746 mimics and HA-ATG7 for 48 h, then infected with NDV. Cells were harvested at 12 h post-infection. Western blot analyses of the NDV-NP protein level, and grayscale analysis performed. (**H**) DF-1 cells were transfected with sicircRNA0857 and miR1709 or miR1746 inhibitors for 48 h, then infected with NDV. Cells were harvested at 12 h post-infection. Western blot analyses of the NDV-NP protein level, and grayscale analysis were performed. (**I**) DF-1 cells were transfected with sicircRNA0857 and HA-ATG3 or HA-ATG7 for 48 h, then infected with NDV. Cells were harvested at 12 h post-infection. Western blot analyses of the NDV-NP protein level, and grayscale analysis were performed. (**J**) DF-1 cells were transfected with pCD25-circRNA0857 for 48 h, then infected with NDV and treated with CQ (25 μM). Cells were harvested at 12 h post-infection. Western blot analyses of the NDV-NP protein level, and grayscale analysis were performed. The MOI of NDV was set at 1 in all the experiments mentioned above. Each bar represents the mean ± standard deviation; *P < 0.05, **P < 0.01, and ***P < 0.001.

Our previous studies have confirmed that NDV infection promotes viral proliferation by inducing cellular autophagy. Therefore, we first investigated the effects of the autophagy-regulating genes ATG3 and ATG7 on NDV replication. The results demonstrated that knockdown of either ATG3 or ATG7 significantly reduced viral copy numbers (Fig. S6A and D), viral titers in the cell supernatant (Fig. S6B and E), and expression of the NDV NP protein (Fig. S6C and F). Given the inhibitory functions of the miR1709/ATG3 and miR1746/ATG7 axes in autophagy induction, we next assessed the impact of miR1709 and miR1746 on NDV replication. Transfection with miR1709 and miR1746 mimics markedly suppressed NDV replication, as demonstrated by reduced viral copy numbers (Fig. 6A), diminished viral titers in the cell supernatant (Fig. 6C), and decreased NDV NP expression (Fig. 6E). Conversely, transfection with miR1709 and miR1746 inhibitors enhanced viral copy numbers (Fig. 6B), viral titers in the cell supernatant (Fig. 6D), and the expression of viral protein (Fig. 6F). Co-transfection of miR1709/miR1746 mimics with ATG3/ATG7 overexpression plasmids showed that ATG3/ATG7 reversed the suppression of NDV replication induced by the miRNA mimics (Fig. 6G). These results confirm that miR1709 and miR1746 regulate viral replication through ATG3 and ATG7, respectively. To further clarify the critical role of the miR1709/ATG3 and miR1746/ATG7 axes in circRNA0857-mediated regulation of viral replication, we analyzed the effects of miR1709 and miR1746 on viral proliferation in circRNA0857-silenced cells. Silencing circRNA0857 alone significantly inhibited NDV replication, an effect that was substantially reversed by transfection with miR1709 or miR1746 inhibitors (Fig. 6H). Importantly, ATG3/ATG7 overexpression also rescued the inhibitory effect on viral replication caused by circRNA0857 knockdown (Fig. 6I), further demonstrating that circRNA0857 functions upstream of the miR1709/ATG3 and miR1746/ATG7 axes. To further confirm that circRNA0857 promotes NDV replication through autophagy, we treated circRNA0857-overexpressing cells with CQ. The results show that CQ treatment significantly attenuated the circRNA0857-mediated enhancement of NDV replication (Fig. 6J). Collectively, these results demonstrate that circRNA0857 enhances viral replication and proliferation by promoting autophagy through the sponging of miR1709 and miR1746.

## DISCUSSION

**Fig 7:**
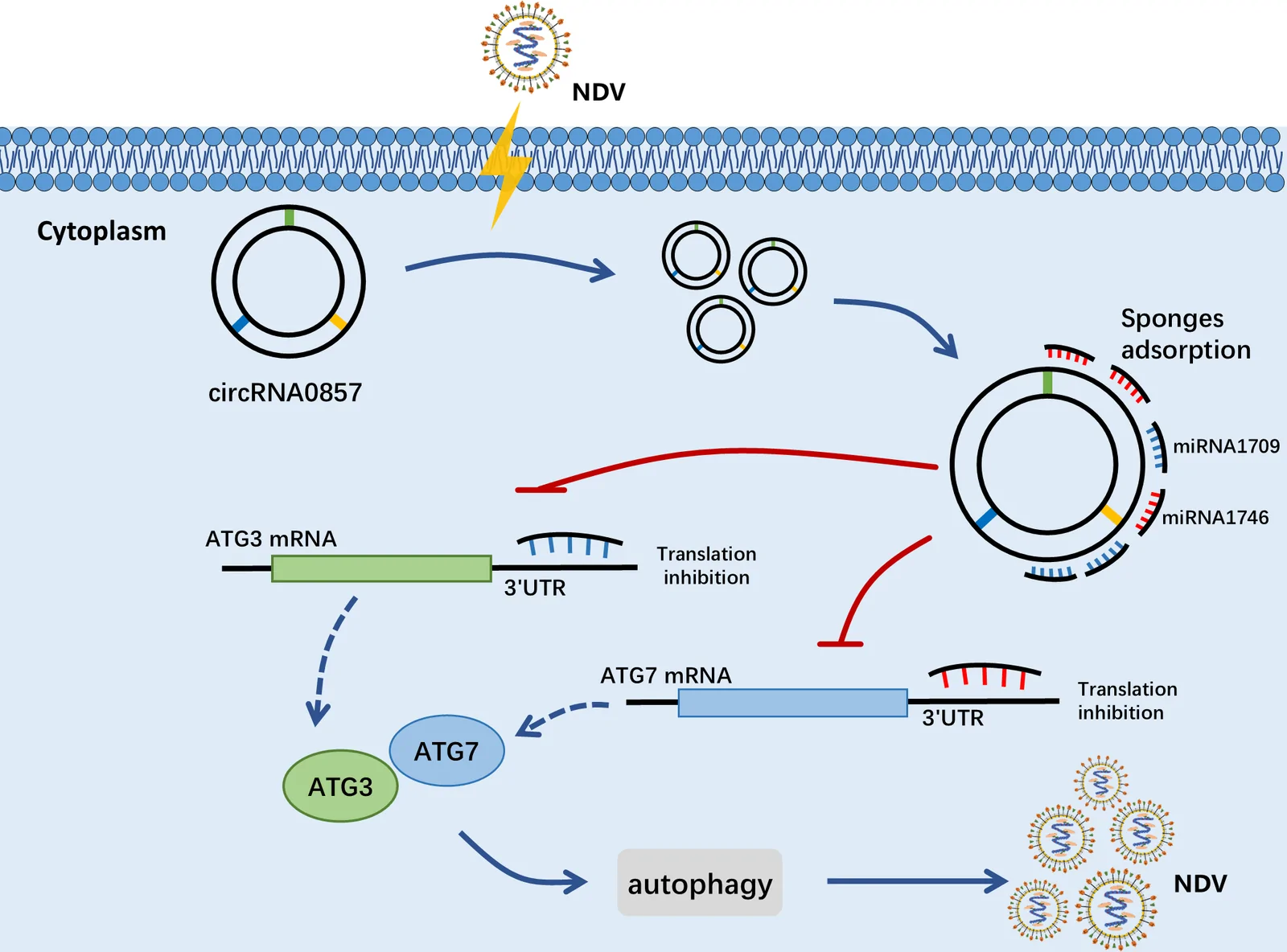
Schematic diagram illustrating the mechanism by which circRNA0857 enhances autophagy to promote NDV replication through the miR1709/ATG3 and miR1746/ATG7 axes. Infection with NDV upregulates the expression of circRNA0857. As a molecular sponge for miR1709 and miR1746, circRNA0857 adsorbs these miRNAs, thereby releasing their inhibitory effects on the autophagy-related genes ATG3 and ATG7. This process promotes autophagy induced by NDV and facilitates viral proliferation.

Currently, many studies focus on the crucial role that ncRNAs, including miRNAs and circRNAs, play in regulating viral infections through complex RNA interaction networks. miRNAs typically suppress gene expression by binding to complementary sequences in target mRNAs, leading to translational repression or transcript degradation. Conversely, circRNAs function as competitive endogenous RNAs that sequester miRNAs, thereby alleviating their inhibitory effects on downstream targets (36). In this study, we demonstrated that NDV infection upregulates circRNA0857 to enhance viral replication. Mechanistically, circRNA0857 acts as a molecular sponge for miR1709 and miR1746, which respectively target the autophagy-related genes ATG3 and ATG7. By adsorbing these miRNAs, circRNA0857 relieves their suppression of ATG3 and ATG7 expression, thereby promoting NDV-induced autophagy and facilitating viral proliferation (Fig. 7). This study is the first to identify the circRNA0857-miR1709/ATG3 and circRNA0857-miR1746/ATG7 regulatory axes in NDV infection, demonstrating how viral exploitation of host ncRNA networks enhances autophagy to promote viral replication.

The roles of circRNAs have been extensively documented in various tumor diseases, where they participate in regulating tumorigenesis, progression, and drug resistance (37–40). In contrast, the roles of circRNAs in viral infections have received relatively less attention. Some studies have demonstrated that circRNAs exert antiviral functions by modulating the host immune system (41, 42). Furthermore, certain viruses have evolved strategies to exploit circRNAs for their own replication (43). In this study, we found that NDV infection altered host circRNA expression profiles, with circRNA0857 being upregulated while most other dysregulated circRNAs were substantially downregulated. Although the upregulation of circRNA0857 was relatively modest, its status as one of the few induced circRNAs, together with the notion that upregulated genes are generally more likely to favor viral replication, prompted us to pursue further investigation. Our findings demonstrate that circRNA0857 promotes NDV replication, albeit to a limited extent. Given the complexity and redundancy of host regulatory networks, this relatively modest effect is not surprising, as individual factors generally exert only limited effects on viral replication. Whether these downregulated circRNAs participate in NDV-host interactions remains unclear; notably, our preliminary knockdown of two downregulated circRNAs did not significantly affect NDV replication. Additionally, the mechanism by which NDV infection upregulates circRNA0857 expression remains to be determined. Previous studies have shown that viral infections can alter circRNA expression through activation of host transcription factors (43), modulation of circRNA biogenesis machinery (44), or global cellular stress responses (45). Further investigation is needed to clarify the precise regulatory events upstream of circRNA0857.

Autophagy, an intracellular degradation mechanism, can be activated by host cells to combat viral infections; however, it can also be exploited by viruses to enhance their replication and dissemination. CircRNA can act as a regulatory factor, playing either a positive or negative role in this process. This study found that circRNA0857 enhances autophagy through the sponge effect of miR1709/1746, thereby creating a favorable environment for NDV replication. This finding aligns with observations that circ_0076631 significantly enhances the replication of Coxsackievirus B3 (46), and circRNA_8521 facilitates Seneca virus A infection by sponging miRNA-324 to regulate LC3A (47). Moreover, the intricate interplay between autophagy and viral replication is highly context dependent, with its role varying between antiviral and pro-viral effects depending on the specific virus, the stage of infection, and the condition of the host cell. As described in this study, ncRNAs play a pivotal role in regulating the balance between host autophagy and viral replication, serving as key modulators in the dynamic relationship between cellular defenses and viral exploitation. Understanding these regulatory mechanisms may provide valuable insights into therapeutic strategies targeting viral infections.

The central mechanism of this study is the ability of circRNA0857 to act as a molecular sponge for miR1709 and miR1746, thereby alleviating their repression of autophagy-related genes ATG3 and ATG7. The specificity of miR1709 and miR1746 in autophagy regulation is particularly noteworthy. While these miRNAs did not alter ATG3 or ATG7 mRNA levels, they significantly reduced protein abundance, indicating post-transcriptional regulation. Interestingly, their effects were context dependent, manifesting only under autophagy-inducing conditions such as NDV infection, nutrient deprivation, or rapamycin treatment. This indicates that miR1709 and miR1746 function as dynamic regulators of autophagy, activated in response to cellular stress rather than basal conditions. Such specificity may prevent excessive autophagy under homeostatic conditions while allowing precise modulation during infection or other stress responses. Functionally, ATG3 functions as an E2 enzyme (ubiquitin-conjugating enzyme), while ATG7 serves as an E1 enzyme (ubiquitin-activating enzyme). Together, they facilitate the covalent binding of LC3-I to phosphatidylethanolamine, forming LC3-II, which localizes on the autophagosome membrane and participates in proautophagosome extension (48). Previous studies have also reported the regulatory roles of circRNAs targeting ATG7. For instance, in pancreatic cancer, circATG7 can upregulate ATG7 expression by adsorbing miR-766-5p, promoting autophagy and cancer progression (49). However, studies focusing on ATG3 as a target gene in circRNA regulation are relatively limited. Additionally, other autophagy-related genes, such as ATG5, have been implicated in circRNA-mediated regulation. For example, circHECW2 competes with miR-30D to release ATG5, promoting epithelial-mesenchymal transition and enhancing Notch1 signaling (50). These findings emphasize the broader regulatory roles of non-coding RNAs in autophagy-related pathways, which are increasingly recognized as critical players in disease progression and cellular stress responses.

In summary, the present study has revealed that NDV infection significantly upregulates the expression of circRNA0857. CircRNA0857 acts as a molecular sponge for miR1709 and miR1746, thereby relieving their inhibitory effects on the target genes ATG3 and ATG7. This regulatory mechanism enhances autophagy, which in turn promotes NDV replication. Our findings elucidate a novel pathway through which autophagy modulates NDV replication, thereby enriching our understanding of the role of circRNA in viral replication regulation.

## MATERIALS AND METHODS

### Cells and viruses

DF-1, BHK-21, A549, and HEK-293T cells were procured from the American Type Culture Collection. The cells were cultured in Dulbecco’s modified Eagle medium supplemented with 10% fetal bovine serum (Gibco, Franklin Lakes, NJ, USA). The NDV Herts/33, Mukteswar, and La Sota strains were sourced from the China Institute of Veterinary Drug Control (Beijing, China). NDV titers were quantified by measuring the median tissue culture infectious dose in DF-1 cells.

### RNA sequencing

Based on whole-transcriptome sequencing data from CEF cells infected with NDV Herts/33, circRNA expression was analyzed using bioinformatic techniques. This analysis was completed by Novogene (Beijing, China). Specific sequencing methods and results are referenced from previous studies (32, 33). Differentially expressed circRNAs (|log2FoldChange| > 1.5) were selected for further verification and screening.

### Antibodies and reagents

A monoclonal antibody against NDV-NP was prepared in our laboratory. Rabbit monoclonal anti-ATG3 (3,415), anti-ATG7 (8,558), and anti-AGO2 (2,897) antibodies were purchased from Cell Signaling Technology (Beverly, MA, USA). Rabbit monoclonal anti-LC3B antibodies were purchased from Abcam (Cambridge, MA, USA). Rabbit monoclonal anti-β-actin (AC006) antibodies were purchased from ABclonal (Wuhan, China). Rabbit monoclonal anti-β-actin (AC006) and anti-HA (AE105) antibodies were purchased from ABclonal (Wuhan, China). Alexa Fluor goat anti-rabbit-488 (A11034) and Alexa Fluor goat anti-rabbit-594 (A11037) antibodies were purchased from Invitrogen (Carlsbad, CA, USA). Drug Rapamycin was purchased from MedChemExpress (HY-10219, Monmouth Junction, NJ, USA). EBSS was purchased from Beyotime (C0213-500 mL, Shanghai, China).

### Reverse transcription and real-time quantitative PCR

Total RNA was extracted using TRIzol reagent (Vazyme). According to the manufacturer’s instructions, the extracted RNA was reverse transcribed using One-Step gDNA Removal and cDNA Synthesis Super Mix (AE311, TransGen Biotech, Beijing, China). Table 1 shows the miRNA-specific reverse primers. Quantitative real-time PCR was performed using a universal blue qPCR SYBR Green Master Mix (AQ601-01-V2, TransGen Biotech, Beijing, China). Mature miRNAs are detected using sequence-specific stem-loop reverse transcription primers, which are complementary only to the 3′ end of the mature miRNA. This ensures that only mature miRNAs, rather than their precursors, are reverse transcribed into cDNA. Subsequently, quantification is performed using SYBR Green. Table S1 shows the quantitative real-time PCR primers.

The relative expression levels of circRNA, miRNA, and mRNA were analyzed using the 2^−△△Ct^ method. β-Actin was used as the internal reference for circRNA and mRNA expression analysis, while U6 served as the internal reference for miRNA. All experiments were performed in triplicate.

### Analysis of the full-length sequence of circRNA

RNA was extracted from DF-1 cells, and the full-length sequence of circRNA0857 was amplified using RT-PCR with rolling circle amplification primers (R: 5′- ACGTGGCTGGGCTTAA - 3′, F: 5′- GCTCGCTAGGACTGTTTC - 3′). The resulting products were ligated into the pMD-19T vector and subjected to Sanger sequencing. By aligning the intermediate sequences of two identical BSJ-S within the same band, the full-length sequence of circRNA0857 was obtained. The UCSC Genome Browser was utilized for alignment to determine the specific exonic or intronic fragments involved in the circularization process. For the identification of circRNA0857’s circularity, divergent primers specific to circRNA0857 BSJ-S (R: 5′ - AATGAACTGCTCGCTAGGACTG - 3′, F: 5′ - AATGAACTGCTCGCTAGGACTG - 3′) and convergent primers (R: 5′ - AATGAACTGCTCGCTAGGACTG - 3′, F: 5’ - CTTTAAGCCCAGCCACGT - 3′) were employed to perform RT-PCR amplification on both cDNA and gDNA from DF-1 cells. Correctly circularized circRNA would yield bands in cDNA amplified with both convergent and divergent primers, while in gDNA, only bands amplified with convergent primers would be present.

### Transfection of plasmids or siRNAs

The pCD25-circRNA0857 was constructed by inserting the Gallus circRNA0857 sequence into the pCD25-ciR plasmid (GENESEED, Guangzhou, China). The pmirGLO-ATG3 3′UTR WT and pmirGLO-ATG7 3′UTR WT were constructed by inserting the 3′UTR sequences of Gallus ATG3 (NM_001278070.2) and Gallus ATG7 (NM_001030592.2) into the pmirGLO plasmid (GENESEED, Guangzhou, China). The HA-ATG3 and HA-ATG7 were constructed by inserting the Gallus ATG3 and ATG7 sequences into the HA plasmid that was preserved by our laboratory. The sequences of the amplification primers for circRNA0857, ATG3 3′UTR, and ATG7 3′UTR are shown in Table 1. pmirGLO-ATG3 3′UTR Mut and pmirGLO-ATG7 3′UTR Mut were synthesized by the company (GENEWIZ). siRNAs targeting endogenous circRNA0857, ATG7, and ATG3 were purchased from GenePharma (Shanghai, China). The siRNA sequences for circRNA0857, ATG3, and ATG7 are shown in Table 1. Mimics and inhibitors of gga-miR-1709, gga-miR-1746, gga-miR-12243-3p, gga-miR-6657-5p, gga-miR-1610, and gga-miR-20b-3p were also sourced from GenePharma (Shanghai, China). Using Lipofectamine 2000 (Thermo Fisher, USA), expression plasmids or siRNAs were transfected into HEK-293T or DF-1 cells according to the manufacturer’s instructions.

### Western blotting

Total protein was extracted from cells using RIPA lysis buffer (Beyotime) supplemented with phenylmethane sulfonyl fluoride. The lysates were then mixed with an appropriate amount of 5× sodium dodecyl sulfate-polyacrylamide gel electrophoresis gels (SDS-PAGE) protein loading buffer (Beyotime) and incubated at 100°C for 10 min to denature the proteins. Subsequently, the denatured samples were subjected to 10% SDS-PAGE. Membranes were then incubated in blocking buffer (5% non-fat milk powder in Tris-buffered saline with 0.2% Tween 20 [TBS-T]), followed by overnight incubation with the corresponding primary antibodies at 4°C. Following that, the membranes were incubated with the corresponding secondary antibody for 2 h at room temperature. β-actin (Abcam) served as an internal control. Finally, protein bands were detected using the ECL detection system (Tanon).

### Dual‐luciferase reporter assay

pmirGLO-circRNA0857 or pmirGLO-vector was co-transfected with six miRNAs, or NC mimics into HEK-293T cells, respectively. Similarly, pmirGLO-ATG3 3′UTR WT or pmirGLO-ATG3 3′UTR Mut were co-transfected with miR1709 or NC mimics into HEK-293T cells. pmirGLO-ATG7 3′UTR WT or pmirGLO-ATG7 3′UTR Mut were co-transfected with miR1746 or NC mimics into HEK-293T cells. After 36 h of incubation, the following experiment was conducted using the TransDetect Double-Luciferase Reporter Assay Kit (FR201-01-V2, TransGen Biotech, Beijing, China) according to the manufacturer’s instructions. Luminescence signals were detected using the BioTek CYTATION5 instrument. Relative luciferase expression was determined as the ratio of firefly to Renilla luciferase activity. All experiments were performed in triplicate.

### Immunofluorescence assay

DF-1 cells were washed thrice with PBS, fixed in 4% neutral formaldehyde for 30 min, and permeabilized with 0.5% Triton X-100 in TBS-T for 15 min. The samples were then blocked with 3% bovine serum albumin for 45 min at room temperature. Subsequently, the cells were incubated with primary antibodies for 2 h at 37°C or overnight at 4°C, followed by incubation with secondary antibodies for 1 h at 37°C. The cells were washed again, and the nuclei were stained with 0.5 mg/mL 4′,6-diamidino-2-phenylindole. The cells were washed thrice with a blocking buffer between and after each incubation step. Finally, cells were visualized by confocal microscopy using a model LSM880 confocal microscope (Carl Zeiss, Jena, Germany). Image analysis was performed using ImageJ software.

### Fluorescent *in situ* hybridization

DF-1 cells were cultured on glass slides and fixed with 4% paraformaldehyde for 15 min, followed by washing with PBS. The cells were then permeabilized at room temperature with 0.1% Triton-100 for 10 min. Pre-hybridization was performed in pre-hybridization buffer at 37°C for 30 min. Synthetic probes circRNA0857, at a concentration of 100 nM, were added to the hybridization buffer along with miRNA1746 or miRNA1709, and hybridization was carried out overnight in a humidified chamber at 37°C. After hybridization, the samples were washed at 42°C with 4 × saline sodium citrate (SSC), 2 × SSC, and 1 × SSC to remove unbound probes. The nuclei were counterstained with 4′,6-diamidino-2-phenylindole and mounted with an anti-fade mounting medium. ImageJ software (NIH, USA) was used for quantitative analysis of the co-localization of circRNA0857 with miRNA1746 and miRNA1709 in the cells. Probes following sequences: circRNA0857 probe: 5′-FAM -ACUAUUUCUCUGCAAUGUUGCCAUGGAAGA-3′; miRNA1746 probe: 5′-CY3-ACCAUGGGACCACAGCUCUGAG-3′; miRNA1709 probe: 5′-CY3-AGUCAGUGCACUCAUCAUUCCA-3′; miRNA6657-5p probe: 5′-CY3-AGAAGAAAACAGAGUAGAAGAUG-3′.

### RIP assay

The Magna RIP RNA Binding Protein Immunoprecipitation Kit (Millipore) was utilized to conduct the RIP assay following the manufacturer’s protocol. DF-1 cells were harvested and lysed using RIP lysis buffer. The cell lysate supernatants were then incubated with magnetic beads conjugated with anti-AGO2 antibodies overnight at 4°C. The RNA samples were associated with the beads and subsequently extracted using the TRIzol reagent. The enrichment of circRNA0857, miR1709, miR1746, and miR6657-5p in the bound protein complexes was analyzed via real-time quantitative PCR.

### Statistical analysis

All data in this study were presented as means ± standard deviation and were determined using GraphPad Prism 8 software (GraphPad Software, Inc.) to perform statistical analysis. Statistical significance was assessed using Student’s *t*-test or one-way analysis of variance, as appropriate. A two-tailed *P* < 0.05 was considered statistically significant, while *P* < 0.01 was considered highly significant.

## Data Availability

The authors confirm that the data supporting the findings of this study are available within the article and its supplemental material. The raw data can be found at the following link: https://doi.org/10.5281/zenodo.21831959.

## SUPPLEMENTAL MATERIAL

Supplemental material jvi.01121-26-s0001.docx Fig. S1 to S6; Table S1.

ASM does not own the copyrights to Supplemental Material that may be linked to, or accessed through, an article. The authors have granted ASM a non-exclusive, world-wide license to publish the Supplemental Material files. Please contact the corresponding author directly for reuse.
