# Supplementary material for: CircRNA0857 acts as an miRNA sponge to promote Newcastle disease virus replication via modulating autophagy
Source: J Virol. 2026 Aug 27;100(9):e01121-26. doi: 10.1128/jvi.01121-26 (PMC13595918; doi:10.1128/jvi.01121-26)
Supplement: Supplementary file 1 [file jvi.01121-26-s0001.docx]

**Fig. S1** **Analysis of the full-length sequence of circRNA0857.** (A) The distribution of the sources of circular RNA in CEF cells infected with NDV under simulated conditions (B) Alignment of the sequence on the UCSC Genome Browser, showing the genomic position and structure of circRNA0857. (C) Identification of the products obtained from the amplification of circRNA0857-specific primers through sanger sequencing, where the repeated sequences represent the BSJ-S sequence of circRNA0857, and the sequence between the two BSJ-S represents the full-length sequence of circRNA0857.

**Fig. S2** **Autophagic flux analysis and validation of miR1709/miR1746 targeting ATG3/ATG7.** (A) DF-1cells were transfected with pCD25-circRNA0857 for 48 hours, NDV was infected and treated with CQ. Western blot analysis of p62 and LC3 protein levels. (B) Gray scale analysis performed. (C) DF-1 cells were infected with NDV, qRT-PCR analyses of the levels of miR1709 and miR1746. (D) DF-1 cells were transfected with mimics or inhibitors of miR1709 or miR1746 for 48 h, then infected with NDV. The mRNA levels of target genes ATG3 and ATG7 by qRT-PCR. The MOI of NDV was set at 1 in all the experiments mentioned above. Each bar represents the mean ± standard deviation; **P* < 0.05, ***P* < 0.01, ns, not significant.

**Fig. S3** **Silencing ATG3 or ATG7 inhibited NDV-induced autophagy.** (A-D) DF-1 cells were transfected with siATG3 or siATG7 for 48 h and then infected with NDV. Western blot analyses of the LC3 and p62 protein levels, and gray scale analysis performed. (E and F) DF-1 cells were transfected with siNC, siATG3 or siATG7, respectively, then infected with NDV, Immunostaining was performed at 18 h post-infection, LC3 spots were observed and quantified by immunostaining. Scale bars: 10 μm. The MOI of NDV was set at 1 in all the experiments mentioned above. Each bar represents the mean ± standard deviation; ***P* < 0.01, ****P* < 0.001.

**Fig. S4** **Autophagy induction activated miR1709/ATG3 and miR1746/ATG7 axes.** DF-1 cells were transfected with mimic or inhibitors of miR1709 or miR1746. (A-D) Cells were treated with or without EBSS at 48 h post-transfection. Western blot analysis of p62 and LC3 protein levels, and gray scaleanalysis performed. (E-H) Cells were treated with or without Rapamycin at 48 h post-transfection. Western blot analysis of p62 and LC3 protein levels, and gray scale analysis performed. Each bar represents the mean ± standard deviation; ***P* < 0.01, ****P* < 0.001.

**Fig. S5** **Under normal conditions, the miR1709/ATG3 and miR1746/ATG7 axes are not activated.** (A-D) DF-1 cells were transfected with mimics or inhibitors of miR1709 or miR1746, cells were harvested at 48 h post-transfection. Western blot analyses of the p62 and LC3 protein levels, and gray scale analysis performed.

**Fig. S6** **Silencing ATG3 or ATG7 inhibited viral replication.** DF-1 cells were transfected with siATG3, siATG7 or siNC, respectively, for 48 h and then infected with NDV. (A and D) Cells were harvested at 0, 6, 12, 18 and 24 h post-infection. The NDV copy number was determined by qRT-PCR. (B and E) Cell culture supernatants were harvested at 0, 6, 12, 18 and 24 h post-infection for the determination of virus titer. (C and F) Cells were harvested at 12 h post-infection, Western blot analyses of NDV-NP protein level, and gray scale analysis performed. The data are from three separate experiments. The MOI of NDV was set at 1 in all the experiments mentioned above. Each bar represents the mean ± standard deviation; **P* < 0.05, ***P* < 0.01, ****P* < 0.001, and ns, not significant.

Table S1 Primers used in this study

| Primers | Application | Sequence (5’-3’) |
| --- | --- | --- |
| gga-miR-1709 | Reverse transcription | GTCGTATCCAGTGCAGGGTCCGAGGTATTCGCACTGGATACGACAGTCAG |
| gga-miR-1746 |  | GTCGTATCCAGTGCAGGGTCCGAGGTATTCGCACTGGATACGACACCATG |
| gga-miR-12243-3p |  | GTCGTATCCAGTGCAGGGTCCGAGGTATTCGCACTGGATACGACGCTCCC |
| gga-miR-6577-5p |  | GTCGTATCCAGTGCAGGGTCCGAGGTATTCGCACTGGATACGACAGAAGA |
| gga-miR-1610 |  | GTCGTATCCAGTGCAGGGTCCGAGGTATTCGCACTGGATACGACCGCCCG |
| gga-miR-20b-3p |  | GTCGTATCCAGTGCAGGGTCCGAGGTATTCGCACTGGATACGACCTGTAA |
| U6 |  | GTCGTATCCAGTGCAGGGTCCGAGGTATTCGCACTGGATACGACAAAAATATGG |
| circRNA0857 F | Clone | CATTAATATTTCTTCTTTCGAATTCTAATACTTTCAGAAATAGTTTGTG |
| circRNA0857 R |  | AGTATGGAGTTGTTAGCTAGGATCCAGTTGTTCTTACCTCTGCAATGTT |
| ATG7 3'UTR F |  | TCGCTAGCAATGTTAACAGTTACTTTAAATATCT |
| ATG7 3'UTR F |  | ACTCTAGACTCAATTAATGCCTAGAAATTGAA |
| ATG3 3'UTR F |  | CGCTAGCCTAAGATAAAAGATCCTCTATCAAG |
| ATG3 3'UTR F |  | ACTCTAGATATACGGGAATGAAAGTGATTTA |
| ATG3 F |  | TGGCCATGGAGGCCCGAATTCatATGCAGAACGTCATCAACACGG |
| ATG3 R |  | gatccccgcggccgcggtaccTTACATTGTAAAGTGCCTTGTGTAGTCA |
| ATG7 F |  | TGGCCATGGAGGCCCGAATTCatATGGCAGCAGTCAGTAATGAGTCC |
| ATG7 R |  | GATCCCCGCGGCCGCGGTACCTCAGACTGTTTCATCATCACTCATGT |
| si circRNA0857 F | Knochdown | GCAGAGAAAUAGUUUGUGATT |
| si circRNA0857 R |  | UCACAAACUAUUUCUCUGCTT |
| si circRNA1675 F |  | CCATACCCAGAACACCAGCUUTT |
| si circRNA1675 R |  | AAGCTGGTGTTCTGGGTATGGTT |
| si circRNA1676 F |  | GCUCAAGGAUACCCAGAUACATT |
| si circRNA1676 R |  | UGUAUCUGGGUAUCCUUGAGCTT |
| si ATG7 F |  | GACAGGUCUAACUUUAUUATT |
| si ATG7 R |  | UAAUAAAGUUAGACCUGUCTT |
| si ATG3 F |  | GACUACACAAGGCACUUUATT |
| si ATG3 R |  | UAAAGUGCCUUGUGUAGUCTT |
| qcircRNA0857 F | qRT-PCR | AAGCTTGCGGCCGCGGCCACCATGCATCGACTCC |
| qcircRNA0857 R |  | GTAGTCAGCCCGGGATCCTCAGATGAGGCTT |
| qATG3 F |  | AGTACCTCACTCCCGTCCTC |
| qATG3 R |  | TTCTCCTGAAGCCCATTGCC |
| qATG7 F |  | GCATGGAGGATGTCGGCT |
| qATG7 R |  | TGATTGTAGTTCATCTCGCCC |
| qβ-actin F |  | AGACATCAGGGTGTGATGG |
| qβ-actin R |  | TCAGGGGCTACTCTCAGCTC |
| qgga-miR-1709 F |  | ACCCGTGGAATGATGAGTG |
| qgga-miR-1709 R |  | TGCAGGGTCCGAGGTATT |
| qgga-miR-1746 F |  | TGGTGTCGTATCCAGTGC |
| qgga-miR-1746 R |  | TGCAGGGTCCGAGGTATT |
| qgga-miR-12243-3p F |  | GGAGCGTCGTATCCAGTG |
| qgga-miR-12243-3p R |  | TGCAGGGTCCGAGGTATT |
| qgga-miR-6577-5p F |  | AGCCGCATCTTCTACTCTGTT |
| qgga-miR-6577-5p R |  | CAGTGCAGGGTCCGAGGTAT |
| qgga-miR-1610 F |  | AACGGGCGGTCGTATCCA |
| qgga-miR-1610 R |  | TGCAGGGTCCGAGGTATT |
| qgga-miR-20-3p F |  | CAGGTCGTATCCAGTGCG |
| qgga-miR-20-3p R |  | TGCAGGGTCCGAGGTATT |
| qU6 F |  | GCTCGCTTCGGCAGCACATATAC |
| qU6 R |  | AGTGCAGGGTCCGAGGTATT |
| qNDV-NP F |  | CAACAATAGGAGTGGAGTGTCTGA |
| qNDV-NP R |  | CAGGGTATCGGTGATGTCTTCT |
| qhuman-β-actin F |  | TGTGGCCGAGGACTTTGATT |
| qhuman-β-actin R |  | CCTGTGTGGACTTGGGAGAG |
